# Epistatic interactions of *CDKN2B*-*TCF7L2 *for risk of type 2 diabetes and of *CDKN2B*-*JAZF1 *for triglyceride/high-density lipoprotein ratio longitudinal change: evidence from the Framingham Heart Study

**DOI:** 10.1186/1753-6561-3-s7-s71

**Published:** 2009-12-15

**Authors:** Ping An, Mary Feitosa, Shamika Ketkar, Avril Adelman, Shiow Lin, Ingrid Borecki, Michael Province

**Affiliations:** 1 Division of Statistical Genomics and Department of Genetics, Washington University School of Medicine (Campus Box 8506), 4444 Forest Park Boulevard, St. Louis, Missouri 63108, USA

## Abstract

Fifteen known type 2 diabetes (T2D) gene variants were assessed for their associations with T2D status in 228 T2D families from the Framingham Heart Study (FHS) Original, Offspring, and Children Cohorts. Bayesian approach was used to test single-single-nucleotide polymorphism (SNP) association followed by logistic regression. Bayesian and logic regression approaches were used to test multiple SNP association searching for the best combinations of variants followed by logistic regression reconfirmation. The significant variants for T2D risk were also tested for their main and interacting effects on triglyceride (TG)/high-density lipoprotein (HDL) ratio change derived from four point measures across time. This slope phenotype was made available using mixed model growth curve approach from 155 T2D families in the FHS Offspring Cohort.

**Results:**

*CDKN2B *rs10811661 (*p *= 0.042), *TCF7L2 *rs4506565 (*p *= 0.004), and JAZF1 rs864745 (*p *= 0.04) were individually associated with risk of T2D (OR = 1.0-2.0; effect size <1%). *CDKN2B *and *TCF7L2 *were found with significant main (*p *= 0.02, 0.01) and interacting (*p *= 0.05) effects for increased (OR = 3.0) risk of T2D. *CDKN2B *and *JAZF1 *were found with significant main (*p *= 0.0002 and 0.034) and interacting (*p *= 0.001) effects on increased (*β *= 0.42) TG/HDL ratio longitudinal change. These interacting effects were independent of effects of age and sex with effect sizes of 0.3-0.4% for risk of T2D or TG/HDL ratio longitudinal change.

**Conclusion:**

These synthetic approaches allowed for successful detection of  *CDKN2B* and *TCF7L2* interacting effect for *T2D* risk and *CDKN2B* and * JAZF1 *interacting effect on *TG/HDL *ratio increase over time among  *T2D *families in the *FHS*. These interacting effects were consistent  in conferring risk of *T2D* or progressive insulin resistance with  modest effect sizes.

## Background

Fifteen replicated gene variants for type 2 diabetes (T2D) have been assessed [1]. While more variants remain to be uncovered, challenge exists in better understanding of the interplay among these variants underneath complex T2D pathophysiology and pathogenesis. These variants were either common with negligible effect or rare with relatively larger effect size [1]. In reality, it is very likely that the individual main effect of each variant is undetectable, but collectively their effect emerges. Concurrent increased triglyceride (TG) and decreased high-density lipoprotein (HDL) were characteristics of subjects with insulin resistance, mostly seen in patients with T2D. The inverse relationship and combined information of these two measures, TG/HDL ratio, represents a single inherited phenotype [2] as a surrogate for insulin resistance [3]. In this analysis, we hypothesized that multiple variants jointly confer T2D susceptibility, and we wanted to examine main and interacting effects of the reported variants in the Offspring Cohort of the Framingham Heart Study (FHS) using different statistical analysis approaches.

## Methods

### Subjects

All participants in the FHS were Caucasians. Their characteristics were described elsewhere [4]. In brief, participants with diabetes comprised 7.59% (28/369), 9.91% (242/2,441), and 2.38% (95/3,997) of the Original, Offspring, and Children Cohorts, respectively. Average age of diagnosis of diabetes at study visit were 66, 57, and 46 years in the three cohorts, respectively. From a total of 6,807 members in 1,157 families, 228 families had members with diabetes (3,217 members, 366 diabetes cases); 46% (1,489/3,217) were men. In the Offspring Cohort, 155 families had members with diabetes (727 members) who had valid fasting TG and HDL measures at 4 time points. In the Offspring Cohort families with member with diabetes, the average age was 33.8, 46.4, 53.3, and 60.2 years, average BMI was 25.2, 26.2, 27.6, and 28.3 kg/m^2^, average TG was 83.7, 108.5, 134.0, and 135.5 mg/dl, average HDL was 51.6, 52.6, 51.6, and 54.5 mg/dl, and average TG/HDL ratio was 1.9, 2.5, 3.1, and 3.0, at Visit 1, 3, 5, and 7, respectively. Every study subject provided written informed consent. The study was approved by Boston University Institutional Review Board.

### Genotyping

Affymetrix 100 k single-nucleotide polymorphisms (SNPs) and genotype annotation resources were described elsewhere [4]. Identical variants or variants in the same intron/exon regions were identified using Affymetrix 500 k SNPs.

### Statistical analysis

Covariates included age, age^2^, and sex. BIMBAM (Bayesian imputation-based association mapping) [5] was used to assess single-SNP effect according to Bayes Factor (BF) and *p*-value (based on 10,000 permutations). This was followed by logistic-GEE (generalized estimating equations) single-SNP test under general and additive assumptions. BIMBAM output corrected *p *for multiple testing. BIMBAM was further used to identify the best multiple-SNP combination with the highest BF. LOGREG [6] was also used to find the best multiple SNP combination as well as parameter estimates. Findings from these two approaches were compared. The best interacting variants may be tested for main and interacting effects using the logistic-GEE approach. Further, TG/HDL ratios at the four visits in the families in the Offspring Cohort with member with diabetes were used to derive TG/HDL ratio change (slope) via the mixed growth curve model. The mixed sandwich estimator approach was used to assess associations between the identified interacting variants and the slope phenotype.

## Results

Single-SNP test results are presented in Table 1 (BIMBAM) and Table 2 (logistic-GEE, general model). Three variants were found to significant under additive assumption: *CDKN2B *rs10811661 (*p *= 0.0011), *TCF7L2 *rs4506565 (*p *= 0.0043), and *JAZF1 *rs864745 (*p *= 0.0402). Multiple SNP test results are also given in Table 1. LOGREG results, consistent with the BIMBAM finding, suggested almost the same best interacting variants. Interaction between *CDKN2B *rs10811661 and *TCF7L2 *rs4506565 was significantly detected using the logistic-GEE model (OR = 3.0, *p *= 0.02 and 0.01 and 0.05 for main and interacting effects). For the slope trait, single-SNP test results were non-significant for *CDKN2B *rs10811661 (*p *= 0.4621), but significant for *TCF7L2 *rs4506565 (*p *= 0.0379) and *JAZF1 *rs864745 (*p *= 0.0349) in the mixed sandwich estimator additive model. Interaction between *CDKN2B *rs10811661 and *TCF7L2 *rs4506565 variants were significant (*β *= 1.6667, *p *= 0.0002, 0.0341, and 0.0012 for main and interacting effects), and it was associated with high slope values, which may be expressed as an increase in insulin resistance across ages.

**Table 1:** Search results for best single and multiple SNP associations with T2D status

Method	Gene	Chr	Position	RAF^a^	Bayes Factor	Rank	*P* ^b^
**BIMBAM**							
rs10811661	*CDKN2A/2B*	9p	22124094	T, 0.82	0.978	1	0.0045
rs4506565	*TCF7L2*	10q	114748339	T, 0.36	0.591	2	0.0154
rs864745	*JAZF1*	7p	28147081	A, 0.52	0.041	3	0.0397
rs17030946	*THADA*	2p	43587013	T, 0.10	0.035	4	0.0771
rs7961581	*TSPAN8-LGR5*	12q	69949369	T, 0.70	-0.161	5	0.1298
rs10946398	*CDKAL1*	6p	20769013	C, 0.33	-0.284	6	0.2382
rs5215	*KCNJ11*	11p	17365206	C, 0.36	-0.292	7	0.0944
rs2793823	*ADAM30*	1p	120239241	G, 0.89	-0.366	8	0.4292
rs10282940	*SLC30A8*	8q	118257007	G, 0.89	-0.381	9	0.4606
rs5018648	*WFS1*	4p	6343719	G, 0.61	-0.457	10	0.2901
rs8050136	*FTO*	16q	52373776	A, 0.41	-0.457	11	0.4008
rs1801282	*PPARG*	3p	12368125	G, 0.11	-0.488	12	0.8047
rs564398	*CDKN2A/2B*	9p	22019547	G, 0.39	-0.517	13	0.5119
rs4402960	*IGF2BP2*	3q	186994381	G, 0.67	-0.601	14	0.4523
rs4607103	*ADAMTS9*	3p	64686944	C, 0.74	-0.613	15	0.8136
SNP pair 1	--	--	--	--	1.76	1, 2	--
SNP pair 2	--	--	--	--	1.064	1, 3	--

**LOGREG**	**Gene**	**Chr**	**Position**	**RAF**	**Beta**	**Mode^c^**	**Logic**
rs864745	*JAZF1*	7p	28147081	A, 0.52	0.03 (tree 1)	Rec	and
rs10811661	*CDKN2A/2B*	9p	22124094	T, 0.82	0.09 (tree 2)	Rec	and
rs4506565	*TCF7L2*	10q	114748339	T, 0.36	0.09 (tree 2)	Dom	and
rs5215	*KCNJ11*	11p	17365206	C, 0.36	0.09 (tree 2)	Dom	or

^a^RAF, risk allele and frequency

^b^Permutation-based *p*-values for single-SNP association tests with T2D status using BIMBAM

^c^Rec, recessive coding; Dom, dominant coding

**Table 2:** Single-SNP association test results for T2D status confirmed by regression analysis

SNP	Gene	Chr	Position	RAF	OR (95% CI)	R^2^	*P* ^a^
rs2793823	*ADAM30*	1p	120239241	G, 0.89	2.0 (0.3-13.0) 2.2 (0.4-14.1)	0.0007	0.4762 0.3905
rs17030946	*THADA*	2p	43587013	T, 0.10	1.2 (0.3-5.2) 0.8 (0.5-1.2)	0.0044	0.793 0.2617
rs4607103	*ADAMTS9*	3p	64686944	C, 0.74	1.1 (0.6-2.1) 1.1 (0.6-2.1)	0.0002	0.6784 0.3905
rs1801282	*PPARG*	3p	12368125	G, 0.11	1.2 (0.1-28.7) 0.9 (0.6-1.3)	0.0004	0.8895 0.625
rs4402960	*IGF2BP2*	3q	186994381	G, 0.67	1.2 (0.8-2.0) 1.2 (0.7-1.9)	0.0014	0.3728 0.5386
rs5018648	*WFS1*	4p	6343719	G, 0.61	1.1 (0.7-1.6) 1.0 (0.6-1.4)	0.0017	0.8127 0.8432
rs10946398	*CDKAL1*	6p	20769013	C, 0.33	1.2 (0.7-2.1) 1.3 (0.9-2.1)	0.0014	0.4013 0.1858
rs864745	*JAZF1*	7p	28147081	A, 0.52	1.5 (1.0-2.3) 1.1 (0.8-1.6)	0.0043	**0.0427^b^** 0.6146
rs10282940	*SLC30A8*	8q	118257007	G, 0.89	1.3 (0.4-4.5) 1.3 (0.4-4.3)	0.0006	0.6785 0.7141
rs10811661	*CDKN2A/2B*	9p	22124094	T, 0.82	2.1 (1.0-4.4) 1.3 (0.6-2.9)	0.0061	**0.0424** 0.4428
rs564398	*CDKN2A/2B*	9p	22019547	G, 0.39	1.1 (0.7-1.9) 1.0 (0.8-1.4)	0.0007	0.5892 0.7728
rs4506565	*TCF7L2*	10q	114748339	T, 0.36	2.0 (1.2-3.1) 1.4 (0.9-2.0)	0.0045	**0.0041** 0.0932
rs5215	*KCNJ11*	11p	17365206	C, 0.36	1.0 (0.6-1.7) 0.7 (0.5-1.0)	0.0039	0.9201 0.0619
rs7961581	*TSPAN8-LGR5*	12q	69949369	T, 0.70	1.1 (0.7-1.8) 1.3 (0.8-2.2)	0.0024	0.6417 0.2377
rs8050136	*FTO*	16q	52373776	A, 0.41	1.1 (0.7-1.7) 0.9 (0.7-1.2)	0.001	0.6854 0.6667

^a^Logistic-GEE regression *p *for genotypes homozygous (line 1) and heterozygous (line 2) for a risk allele with genotype homozygous for a non-risk allele as reference.

^b^Bold font indicates significant *p*-values.

## Discussion

Our finding of *TCF7L2 *intron 3 variant for T2D is in line with a previous FHS genome-wide association study report [7] and a good number of replicating reports across large and independent study cohorts. Evidence of *CDKN2B *near/promoter and *JAZF1 *intron 1 variants for T2D risk was also found. We thought the T2D families in FHS may contain an enriched genetic component for SNP association assessment of T2D gene variants. Currently, functions of these two genes are yet not clear, but as is the case for *TCF7L2 *and other confirmed T2D genes [1], they may be directly or indirectly involved in pancreatic *β*-cell failure, insulin resistance, and lipid toxicity. Our finding of interactions among the three variants for T2D risk was interesting and novel, which may help disentangle underlying gene pathways and further our understanding of T2D etiology and pathophysiology.

Correction for multiplicity to minimize the false-positive declaration rate seems to be less sensitive for this analysis for two reasons. First, we aimed to identify and evaluate the best combination of SNPs from confirmed T2D variants. Second, the FHS T2D status information was obtained at the initial visit of the study, and 'new' T2D cases may have remained in the 'non-diabetic' control group after decades of follow-up. This would bias our association findings towards reduction of type 1 error. The results of BIMBAM were consistent with those of logistic-GEE analyses for the single-SNP main effect; likewise, BIMBAM and logic regression results for multiple-SNP joint/interacting effect were also consistent. These synthetic approaches apparently worked well in association detection for both main and joint effects of candidate gene variants with prior knowledge.

This 500 k SNP genotype data allowed us to examine 15 of the 18 confirmed exact variants (genotypic imputation was not applied). While reproducibility may be cohort-specific and sensitive to sample heterogeneity and size, it was a bit surprising that we could only identify three meaningful variants. It is very possible that this was due to the presence in the control groups of persons with diabetes who had not received a clinical diagnosis. We did not use a transmission-disequilibrium test-like FBAT program for this analysis simply because we wanted to maximize the sample size by using all available family information. We did not correct the data for potential effect of body max index because obesity coexisting with T2D is very likely rooted in the same pathway genes among most T2D patients, at least in most Western countries.

We were excited to find that the identified interacting variants also affected the TG/HDL ratio. Using the ROC/AUC (receiver operator characteristic/area under the curve) model, we found that it was an imperfect predictor of T2D with best sensitivity and specificity of approximately 75%. Applying a mixed growth curve approach to model longitudinal multiple measures of the TG/HDL ratio proved useful. The derived slope phenotype represented the TG/HDL ratio change across decades of observation time. This approach is advantageous in handling missing data points, as well as remote values, especially in intervention and interaction studies. Genetic heritability of the slope phenotype was 20%, and these three variants were individually and interactively associated with an increase of the slope phenotype. Previously, it was thought that the *TCF7L2 *variant was only associated with *β*-cell function failure but not insulin resistance [1]. Our current finding suggests that *TCF7L2*, together with *JAZF1 *variants, may indirectly impart insulin resistance through joint modulation of lipid metabolism.

In conclusion, using the FHS T2D family data and different statistical approaches, we found variants in the *TCF7L2*, *CDKN2B*, and *JAZF1 *genes were independently and interactively associated with increased T2D risk and the TG/HDL ratio change across ages. For the remaining T2D risk conferring gene variants, we did not find significant association evidence in this analysis.

## List of abbreviations used

AUC: Area under the curve; BF: Bayes factor; BIMBAM: Bayesian imputation-based association mapping; GAW16: Genetic Analysis Workshop 16; GEE: Generalized-estimating equations; HDL: High-density lipoprotein; TG: Triglyceride; T2D: Type 2 diabetes; FHS: Framingham Heart Study; ROC: Receiver operator characteristic; SNP: Single-nucleotide polymorphism;

## Competing interests

The authors declare that they have no competing interests.

## Authors' contributions

PA, MF, IB, and MP drafted the manuscript. SK, AA, and SL performed the statistical data analysis. All authors read and approved the final manuscript.
